# Stocks of carbon and nitrogen and partitioning between above- and belowground pools in the Brazilian coastal Atlantic Forest elevation range

**DOI:** 10.1002/ece3.41

**Published:** 2011-11

**Authors:** Simone A Vieira, Luciana F Alves, Paulo J Duarte-Neto, Susian C Martins, Larissa G Veiga, Marcos A Scaranello, Marisa C Picollo, Plinio B Camargo, Janaina B do Carmo, Eráclito Sousa Neto, Flavio A M Santos, Carlos A Joly, Luiz A Martinelli

**Affiliations:** 1 Laboratório de Ecologia Isotópica—CENA/USPSP, Brazil; 2 NEPAM/UNICAMPSP, Brazil; 3 INSTAAR, University of ColoradoBoulder, Colorado; 4 Instituto de BotânicaSão Paulo, SP, Brazil; 5 Unidade Acadêmica de Garanhuns, Universidade Federal Rural de PernambucoGaranhuns-PE, Brazil; 6 Universidade Federal de São Carlos, Campus de SorocabaSorocaba, Brazil; 7 Instituto de Biologia/UNICAMPSP, Brazil

**Keywords:** Atlantic forest, carbon stocks, nitrogen stocks, elevation range, montane tropical forest

## Abstract

We estimated carbon and nitrogen stocks in aboveground biomass (AGB) and belowground biomass (BGB) along an elevation range in forest sites located on the steep slopes of the Serra do Mar on the north coast of the State of São Paulo, southeast Brazil. In elevations of 100 m (lowland), 400 m (submontane), and 1000 m (montane) four 1-ha plots were established, and above- (live and dead) and belowground (live and dead) biomass were determined. Carbon and nitrogen concentrations in each compartment were determined and used to convert biomass into carbon and nitrogen stocks. The carbon aboveground stock (C*_AGB_*) varied along the elevation range from approximately 110 to 150 Mg·ha^−1^, and nitrogen aboveground stock (N*_AGB_*), varied from approximately 1.0 to 1.9 Mg·ha^−1^. The carbon belowground stock (C*_BGB_*) and the nitrogen belowground stock (N*_BGB_*) were significantly higher than the AGB and varied along the elevation range from approximately 200–300 Mg·ha^−1^, and from 14 to 20 Mg·ha^−1^, respectively. Finally, the total carbon stock (C*_TOTAL_*) varied from approximately 320 to 460 Mg·ha^−1^, and the nitrogen total stock (N*_TOTAL_*) from approximately 15 to 22 Mg·ha^−1^. Most of the carbon and nitrogen stocks were found belowground and not aboveground as normally found in lowland tropical forests. The above- and belowground stocks, and consequently, the total stocks of carbon and nitrogen increased significantly with elevation. As the soil and air temperature also decreased significantly with elevation, we found a significantly inverse relationship between carbon and nitrogen stocks and temperature. Using this inverse relationship, we made a first approach estimate that an increase of 1°C in soil temperature would decrease the carbon and nitrogen stocks in approximately 17 Mg·ha^−1^ and 1 Mg·ha^−1^ of carbon and nitrogen, respectively.

## Introduction

It has been recognized now that terrestrial ecosystems have substantial effects on climate regimes, in addition to being strongly affected by climate (Cox et al. 2000; Yurova et al. 2010). Tropical forests are one of the most important biomes on earth, contributing approximately 36% of the net carbon exchange between atmosphere and terrestrial vegetation (Melillo et al. 1993), and accounting for a significant fraction of the total carbon and nitrogen stocks both in their biomass and soil (Brown and Lugo 1984; Dixon et al. 1994; Brown et al. 1995; Phillips et al. 1998; Houghton 2005; Houghton et al. 2009). It has recently been shown that tropical forests, such as the Amazon, may suffer profound changes in its structure and functioning with changes in precipitation and temperature (Harris et al. 2008).

The Atlantic Forest domain (Morellato and Haddad 2000; Oliveira–Filho and Fontes 2000) is a South American tropical biome of immense structural complexity harboring some of the most diverse and biologically unique forest ecosystems on earth (Wilson 1992; Davis et al. 1997; Myers et al. 2000). Prior to 1850, the Atlantic Forest was one of the largest American tropical forests, originally covering an area of ca. 1.5 million km^2^ (Ribeiro et al. 2009); today it is one of the most threatened (Myers et al. 2000; Laurance 2009) and human-altered ecosystems in the tropics (Por 1992; Dean 1997; Metzger et al. 2009), restricted to only 12% of its original area (Ribeiro et al. 2009). Within the tropical Atlantic Forest, much of the intact forest areas are found in a coastal region of Brazil, mainly in mountains of the southeast, where relief has limited their use to agricultural purposes and urbanization (Ranta et al. 1998; Resende et al. 2002; Silva et al. 2007; Ribeiro et al. 2009).

Although most of the biomass stock of the Atlantic Forest has been removed in the last 150 years (Dean 1997; Drummond 2004), there are scarce data on the amount of carbon stored in soils and plants of this tropical forest (Tiepolo et al. 2002; Rolim et al. 2005; Alves et al. 2010; Coutinho et al. 2010), as well as on how biogeochemical patterns have been altered by different land-use strategies, or how these might influence reforestation efforts (Domingos et al. 1998; Moraes et al. 1999; Villela et al. 2006). Detailed estimates of carbon and nitrogen stocks in remaining forest areas can provide new insights for better conservation, as well as regeneration practices of the Atlantic Forest (Alves et al. 2010). Additionally, it would improve our understanding about mechanisms that sustain and regulate biodiversity, as well as processes controlling the structure and function of these forests. Moreover, in times of climate change, the potential impacts of global climate change on ecosystem productivity and biogeochemical cycling is of major concern (Melillo et al. 1993). Determining how ecosystem functioning might be altered is critical to understanding the full consequences of global climate change (Millennium Ecosystem Assessment, 2005).

Marengo et al. (2009) predict a warmer and drier climate for southern Brazil in the next 50 years that may considerably alter the functioning of these important ecosystems, influencing the carbon sequestration capacity of soil and live biomass through differential effects on plant productivity (Bonan 2008) and associated debris supplied to the soil and on decomposition (Meier and Leuschner 2010). As a consequence, the partitioning of carbon in pools with rapid and slow turnover can also be changed (Norby et al. 2002; Meier and Leuschner 2010) and can affect the soil compartment, which plays an important role in ecosystem processes with potential feedback on atmospheric CO_2_ concentration and climatic change (Trumbore et al. 1996). Hence, studies in Atlantic forest sites along elevational gradients are particularly important, because they may serve as a means to determine the effects of climate on ecosystem-level processes (McGroddy and Silver 2000), since decreases in air temperature relative to lower elevations are intrinsic to all mountainous areas.

Elevational gradients provide a useful basis for studying the influence of environmental factors such as light, temperature and precipitation on important forest ecosystem functions, and carbon and nutrient cycling processes (Raich et al. 2006; Moser et al. 2008; Malhi et al. 2010; Salinas et al. 2010). However, few studies have examined carbon and nutrient partitioning along elevational gradients in the tropics and quantified its relationship to climate (Kitayma and Aiba 2002; Leuschner et al. 2007; Moser et al. 2008; Girardin et al. 2010; Malhi et al. 2010). In the present study, above- (live and dead vegetation) and belowground (soil organic matter and roots) carbon and nitrogen pools were quantified for forest sites distributed along an elevational range of 1000 m in the coastal Atlantic Forest of southeast Brazil. The elevational range chosen for this study is peculiar, as soil type, parental age material, and precipitation do not vary greatly along the elevation range (Martins, 2010; Sousa Neto et al. 2011). The lack of such confounding attributes makes it easier to detect the effects of air and soil temperature in major biological processes (such as rates of photosynthesis and respiration, decomposition, microbial activity) affecting C and N pools along the elevation. The specific goals of this study are: (1) to quantify the carbon and nitrogen pools in soil and biomass along an elevation range in the coastal Atlantic Forest of Brazil, and their correlation with temperature, (2) to evaluate how the patterns between above- and belowground carbon and nitrogen allocation differ along the elevational range, and (3) to evaluate how the patterns of carbon and nitrogen allocation differ between the Atlantic Forest and the Amazon forest.

## Materials and Methods

### Study site

We investigated the carbon and nitrogen pools in biomass and soil in tropical forest stands across an elevation range in the Atlantic Forest plots. The study area is a mountainous landscape with steep slopes included in the “Serra do Mar” biogeographical region (Silva et al. 2007; Ribeiro et al. 2009).

We selected three sites along a range of elevations located in the “Serra do Mar” State Park, which belongs to the State of São Paulo, SE Brazil. This natural park contains the largest continuous fragment of Atlantic Forest in Brazil occurring mainly along the coastal mountains of the state encompassing, according to Ribeiro et al. (2009), an area of approximately 1 million hectare.

The sampling areas are located in the municipalities of Ubatuba and São Luis do Paraitinga, (23^o^34′S, 45^o^02′W and 23^o^17′S, 45^o^11′W), which in turn are located on the north coast of São Paulo State between 100 and 1100 m above sea level (asl), respectively. The physiognomies of the Atlantic Forest in coastal zones are classified according to the rainfall and temperature of their occurrence, which in turn, is based on latitude and altitude (Veloso et al. 1991; Oliveira Filho and Fontes 2000). At lower altitude, forests are classified as Lowland Moist Dense Forest (<300 m asl and hereafter referred to as lowland forest), followed by Submontane Moist Dense Forest (300–700 m asl and hereafter referred to as submontane forest) and Montane Moist Dense Forest (>700 m asl and hereafter referred to as montane forest).

The historical average annual rainfall is approximately 3000 mm (with the lowest precipitation in June: 87 mm) and the yearly average temperature is 22°C measured at a meteorological station located 220 m asl. In the montane forest, the regional climate is classified as Cwa (temperate-warm climate type, with summer rains and hot summer), according to Köppen (1948). The historical average annual precipitation measured in a meteorological station located at 760 m asl was 2300 mm, and in the dry season (July and August) the precipitation is not less than 60 mm, and the yearly average temperature is 17°C (Salemi 2009).

The predominant lithology is mainly composed of a crystalline basement (Pre-Cambrian) of the “Coastal Complex,” with predominance of metamorphic (gneisses and migmatite) and granitic rocks (IPT 2000). Banks of sedimentary rocks are also observed on the “Coastal Plane” (IPT 2000).

Soils in lowland, submontane, and montane forest sites are classified as Inceptisol (United States Department of Agriculture taxonomy) with more than 50% sand content (Martins et al. in review). These soils have a high sand content in common, with low pH, low phosphorus concentration, low sum of bases, and high aluminum saturation.

The relief varies along the elevation gradient. The lowland forest is inserted in wavy and scarped relief with moderately steep slopes, with large rocks (rocks over 50-cm diameter) covering around 40% of the surface. The relief at the submontane level is characterized by a mountainous relief with the steepest slopes among the sample sites. In the montane forest, the relief is less steep than in the submontane since the peak of the mountain is reached, but still moderately steep slopes predominate.

### Field sampling

In the Atlantic Forest, the aboveground carbon and nitrogen stock was determined according to inventory taken in permanent plots established by Project “BIOTA/FAPESP-Gradiente Funcional” (proc. 03/12595–7). All data used in this work were generated in the project mentioned above, and some of them were already published. Aboveground biomass (AGB) was published by Alves et al. (2010); litter and fine roots stocks by Sousa Neto et al. (2011); and soil stocks by Martins 2009.

**Figure 1:**
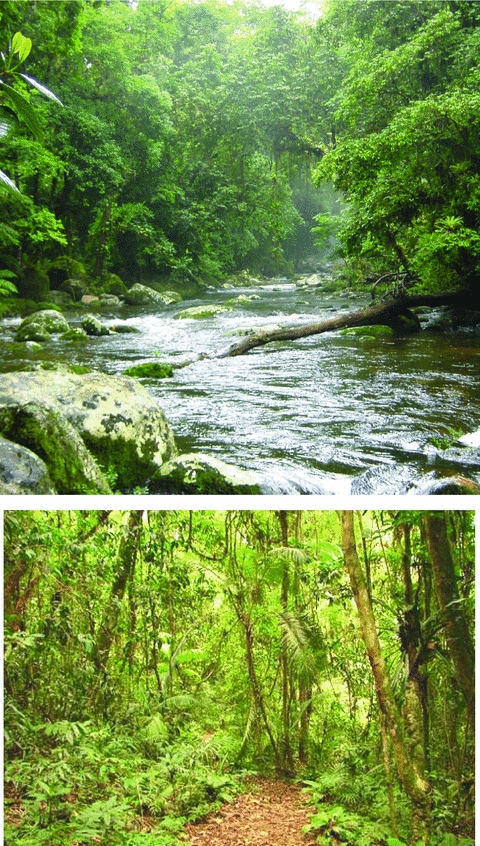
Pictures of the study sites. Upper panel: stream of the montane Atlantic Forest and lower panel: local view of one of our plots at the montane Atlantic Forest.

Four 1-ha plots were established at the lowland, submontane, and montane forests (Fig. 1, Table 1). In all plots, forest structure data and tree, ferns and palms species composition were determined. All individual stems with diameter at breast height (DBH) higher than 10 cm were identified (including trees, palms, and tree ferns), and its DBH and height were measured. For more details on the forest structure see Alves et al. (2010).

**Table 1:** Forest type, 1-ha plot codes, altitude of each plot, number of stems per hectare, and aboveground biomass (AGB)

Forest type	Plot code	Altitude (m)	Soil Temperature (°C)	Stem· ha^−1^	AGB (Mg·ha^−1^)
Lowland	B	46	23.0	597	211
	C	56	22.9	643	190
	D	64	22.9	584	181
	E	76	22.8	617	222
Submontane	G	187	20.6	688	233
	H	209	20.5	691	224
	I	350	19.6	1023	257
	J	372	19.4	870	260
Montane	K	1027	15.0	791	244
	L	1044	14.9	847	323
	M	1050	14.9	834	242
	N	1070	14.8	851	278

The aboveground live carbon and nitrogen stocks were estimated based on data of aboveground live biomass (AGLB) calculated by Alves et al. (2010) (Table 1). Carbon and nitrogen concentrations for trunks were obtained by sampling 30 individuals at each elevation. These individuals were chosen among the most common species in three DBH classes: 10– 30 cm, 30–50 cm, and above 50 cm.

For aboveground leaf biomass, we used the relationship between the amount of leaves in the canopy (P) and annual litterfall (Q) (P/Q = 1.25), proposed by Grubb (1977), also used by Cunha et al. (2009) in a Montane Atlantic Forest.

The belowground live biomass (root) was estimated according to the model proposed by Cairns et al. (2009) (equation 1), based on AGLB.
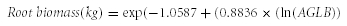
(1)where: AGLB is the total aboveground live biomass (tree + palm + tree fern) (Alves et al. 2010).

Data from carbon and nitrogen stocks in dead fine root biomass (0–20 cm) were obtained from Sousa Neto et al. (2011), who worked in the same areas.

### Coarse wood debris biomass

Dead biomass, or coarse wood debris (*CWD*), was divided into two categories: standing *CWD* (snags) and fallen *CWD*. Standing *CWD*, defined as dead trees standing or at an angle higher than 45^o^, had their DBH and height measured in each permanent plot. The snag mass was calculated from the product of measured volumes (Chambers et al. 2000; Palace et al. 2007) and of *CWD* density (Keller et al. 2004). We used a four-group decay classification system (adapted from Keller et al. 2004) and the associated densities for each decay class for mass calculations (Table 2).

**Table 2:** Mean wood density (gcm^−3^) for four-decay class for Standing *CWD* and five-decay class of fallen *CWD* and medium (5–10 cm) and small (2–5 cm) debris

	g cm^−3^	
	
Decay class	Standing *CWD*	Fallen *CWD*
1	0.51	0.40
2	0.42	0.30
3	0.36	0.22
4	0.30	0.19
5		0.14
Medium (5–10 cm)		0.28
Small (2–5 cm)		0.21

Fallen *CWD* was sampled using the line-intercept method, also known as planar intercept sampling. Three 100-m sampling lines were set parallel to the length axis of the permanent plot, separated from each other by 30 m. The sampling line was then divided into 10-m segments in which the diameter of *CWD* (≥7.5 cm) that crossed the sampling line was measured. In every 5th segment, *CWD* greater than 2 cm in diameter was measured, but decay classes were not recorded for the 2–7.5 cm category. *CWD* volumes were estimated using formulas in Van Wagner (1968), and biomass was estimated using decay class estimates (Table 2).

### Forest floor and soil collection

The litter stock was estimated by Sousa Neto et al. (2011). Briefly, the litter layer on the floor of the Atlantic Forest was sampled every month for a year using traps with an area of 0.25 m^2^ (0.5 × 0.5 m). Thirty traps were used in each of the three elevations. The traps were arranged randomly in the plots and the material collected was wrapped in paper bags and sent to the laboratory where they were dry processed and analyzed. The value presented here represents the average from 1 year of sampling.

For soil carbon and nitrogen stocks, we used the data of Martins et al. (in review). Briefly, samples were taken for 16 soil profiles in each plot (two permanent plots by elevation position). These plots were set 30 m apart from each other and sampled to 100 cm depth. In order to determine carbon and nitrogen concentrations, soil samples were taken with cores at the following depths: 0–5; 5–10; 10–20; 20–30; 30–40; 40–50; 50–75; 75–100 cm. Four pits in each of the plots were sampled to 100-cm depth for analysis of bulk density. Soil-sample forest sites were sieved to <2 mm, then homogenized and split into a smaller subsamples that were handpicked to remove fine roots and charcoal.

### Carbon and nitrogen concentrations and pools

The carbon and nitrogen pools in the biomass of each stand were calculated by multiplying the mean concentration of each component by its respective mass. We estimated carbon and nitrogen pools in the following aboveground compartments: tree trunks and leaves, palm, ferns, snags, *CWD*, and forest litter. We also estimated carbon and nitrogen pools in the following belowground compartments: soil organic matter and roots.

As most of the AGLB is located in tree trunks (Martinelli et al. 2000), we randomly selected approximately 30 trees (DBH > 10 cm) in each elevation and sampled the trunk of these trees by coring in order to determine their carbon and nitrogen concentrations. For aboveground leaves, we sampled approximately 120 trees in each elevation, and for litter, we analyzed samples from 90-litter collectors placed at each elevation level.

To determine the carbon and nitrogen concentration in *CWD* (standing and fallen), we randomly selected approximately 28 trunks, seven from each one of the four-group decay categories, and sampled their trunk. The sample was dry, homogenized, and sieved for chemical analysis.

In order to compare our data with Amazon forest data, we converted data from Amazon biomass in carbon and nitrogen stock for AGLB multiplying biomass by carbon and nitrogen concentration of 47.4% and 0.38%, in that order (Martinelli et al. 2000); and for *CWD* by 43.6% and 0.58%, respectively, and for litter layer by 45.8% and 1.93%, respectively (Ometto et al. 2006).

Leaves and fine litter samples were oven dried at 60°C. After the drying process, samples were grinded in a Wiley mill to a fine powder. Woody samples were also oven dried at 60°C and grinded in an electric burr grinder. Carbon and nitrogen concentrations were determined by an elemental analyzer (EA 1110 CHNS from Carlo Erba Instruments, Milan, Italy). Throughout the test, we used the following nomenclature. Carbon (C*_AGLB_*) and (N*_AGLB_*) nitrogen stocks associated with AGLB are the sum of the stocks of tree, ferns, and palm. Carbon (C*_AGDB_*) and nitrogen (N*_AGDB_*) aboveground dead stocks are the sum of *CWD*, snags, and litter layer. Carbon (C*_AGB_*) and nitrogen (N*_AGB_*) aboveground stocks are the sum of C,N*_AGLB_*+ C,N*_AGDB_*. Carbon (C*_BGB_*) and nitrogen (N*_BGB_*) belowground stocks are the sum of the stocks of soil, fine roots, and roots. The carbon (C*_ECOS_*) and nitrogen (N*_ECOS_*) ecosystem stocks are the sum of C,N*_AGB_*+ C,N*_BGB_*.

### Statistical analyses

We tested the normality of the data distribution for carbon and nitrogen concentrations. As the carbon and nitrogen followed a normal distribution according to the Kolmogorov–Sminorv test, we used parametrical tests in our analysis. Analysis of variance (ANOVA) followed by a post hoc Tukey's Honest test for unequal variance was used to determine differences in carbon and nitrogen stocks along the elevation range. Statistical analyses were performed by Statistica 9.0 software (Stat Soft, Inc. 1984 –2004, Tulsa, OK, USA). Differences at the 0.05 level were reported as significant.

We used a maximum likelihood method to fit a linear regression between carbon and nitrogen stocks in each 1-ha plot and mean annual soil temperatures (Table 1). These soil temperatures were measured once a month at random points along a 30-m transect during soil gases sampling (Sousa Neto et al. 2011). Lower limit (LL) and upper limit (UL) of 95% confidence interval was obtained by bootstrap interaction using the statistical software PopTools version 2.7.5.

## Results

Table 3 summarizes the vegetation carbon and nitrogen contents used here in order to estimate carbon and nitrogen stocks. Most of the AGLB is allocated in tree trunks (Alves et al. 2010). Although the AGLB increased significantly with altitude (Alves et al. 2010), the carbon and nitrogen concentrations in trunks were not statistically different among altitudes (Table 3). On the other hand, soil carbon and nitrogen contents increased significantly with altitude (Martins et al. in review).

**Table 3:** Average carbon and nitrogen concentrations followed by the number of samples (*N*) in vegetal tissues and soil organic matter integrated to 1-m depth in each elevation. For *CWD*, concentrations were determined in different classes of decomposition and not by elevation (see text for details)

	*N* (%)	C (%)	*N*
	Leaves		
Lowland	2.46	45.0	153
Submontane	2.30	45.9	158
Montane	2.61	45.8	183
		Trunk	
Lowland	0.34	46.4	30
Submontane	0.54	45.7	30
Montane	0.52	45.2	31
	Coarse wood debris		
DC1	0.26	47.0	07
DC2	0.32	46.1	07
DC3	0.34	46.1	07
DC4	0.35	45.1	07
	Litter layer		
Lowland	2.07	47.7	264
Submontane	1.60	45.9	102
Montane	1.72	48.0	266
	Soil organic matter		
Lowland	1.53	0.11	256
Submontane	1.74	0.14	256
Montane	2.44	0.18	256

### Carbon and nitrogen stocks in forest biomass and soil

C*_AGLB_* varied between approximately 94 and 127 MgC·ha^−1^ and increased with elevation, with the carbon stock at the lowland forest not being statistically different than at the submontane forest, but significantly lower than the montane forest (*F*_2,9_ = 6.45, *P* = 0.0119) (Table 4). This trend of increasing carbon stocks with altitude followed the AGLB that also increased significantly with altitude (Alves et al. 2010).

**Table 4:** Average stocks of carbon (Mg·ha^−1^) in AGLB (trees, palms, tree ferns, and leaves), AGDB (Snags, Fallen, and Litter layer), and BGB (Fine, Roots, Soil not showed) sampled along the altitudinal range of tropical moist forest (Atlantic Forest, Brazil). Numbers below the average are standard deviations (*n* = 4). Numbers between brackets below the standard deviations are the lower limit (LL) and upper limit (UL) of 95% confidence interval was obtained by bootstrap interaction

	Live biomass					Dead biomass				Belowground			
				
Site	Trunk	Leaf	Palm	Fern	AGLB	Snags	*CWD*	Litter	AGDB	Fine root	Root	BGB	Total TC
Lowland	92.06	1.13	1.04	0.05	94.28	0.67	9.95	4.00	14.62	1.94	17.44	206.38	315.27
	8.71	0.36	0.35	0.04	8.74	0.34	2.68	0.00	2.69	0.00	1.44	1.44	8.81
					(86/101)							(205/207)	(306/322)
Sub-montane	109.36	1.98	1.85	0.02	113.21	1.52	9.33	3.54	14.39	1.86	20.68	256.54	384.14
	7.64	0.89	0.85	0.02	8.74	0.81	1.45	0.00	1.56	0.00	1.34	1.34	10.19
					(105/121)							(255/258)	(375/392)
Montane	118.66	4.14	3.72	0.20	126.72	2.28	16.85	2.64	21.77	5.52	22.76	309.28	457.77
	17.85	1.15	0.98	0.17	16.43	0.56	7.62	0.00	8.06	0.00	2.79	2.79	20.23
					(114/144)							(307/312)	(446/478)

C*_AGDB_* was not different between elevations, ranging from approximately 14 to 22 MgC·ha^−1^. The average contribution along the elevation range of C*_CWD_*–C*_AGDB_* was approximately 70%. The litter layer accounted for about 20% of the C*_AGDB_*, while snag represented only 10% (Table 4). The relative contribution of C*_AGDB_* to C*_ECOS_* varied between 4% and 5% along the elevation range.

C*_BGB_* varied from approximately 200 to 300 Mg·ha^−1^, increasing significantly with elevation (*F*_2,9_ = 2718, *P* < 0.0001). Most of the BGB is composed of soil organic matter and only a small proportion is accounted for by roots and fine roots (Table 4).

Finally, C*_ECOS_* increased significantly with elevation (*F*_2,9_ = 103, *P* < 0.0001), being smallest at the lowland site (∼315 Mg·ha^−1^), increasing to approximately 380 Mg·ha^−1^ at the submontane, and reaching approximately 460 Mg·ha^−1^ at the montane forest (Table 4).

Nitrogen stocks followed a similar pattern to that observed for carbon stocks along the elevation range for most of the compartments. N*_AGLB_* ranged from approximately 0.8 to 1.6 Mg·ha^−1^ (*F*_2,9_ = 53.7, *P* < 0.0001); 0.22 to 0.34 Mg·ha^−1^ for N*_AGDB_* (*F*_2,9_ = 0.63, *P* = 0.5533); and 14 to 20 Mg·ha^−1^ N*_BGB_* (*F*_2,9_ = 1810, *P* < 0.0001) (Table 5). An important difference was that the N*_AGLB_* was statistically different among all elevation ranges. The N*_ECOS_* (*F*_2,9_ = 493, *P* < 0.0001) was similar and followed the same pattern of N*_BGB_*, since the soil nitrogen stock comprises approximately 90% of the N*_ECOS_*.

**Table 5:** Average stocks of nitrogen (Mg·ha^−1^) in AGLB (trees, palms, tree ferns, and leaves), AGDB (Snags, Fallen, and Litter layer), and BGB (Fine, Roots, Soil not shown) sampled along the altitudinal range of tropical moist forest (Atlantic Forest, Brazil). Numbers below the average are standard deviations (*n* = 4). Numbers between brackets below the standard deviations are the lower limit (LL) and upper limit (UL) of 95% confidence interval was obtained by bootstrap interaction

	Live biomass					Dead biomass				Belowground			
				
Site	Trunk	Leaf	Palm	Fern	AGLB	Snags	*CWD*	Litter	AGDB	Fine root	Root	BGB	Total TC
Lowland	0.67	0.06	0.01	0.00	0.75	0.00	0.06	0.17	0.24	0.08	0.14	13.72	14.71
	0.06	0.02	0.00	0.00	0.07	0.00	0.02	0.00	0.02	0.00	0.01	0.01	0.07
					(0.93/1.03)							(13.71/13.73)	(14.64/14.75)
Sub-montane	1.29	0.10	0.02	0.00	1.41	0.02	0.20	0.12	0.34	0.07	0.17	18.14	19.89
	0.09	0.04	0.01	0.00	0.13	0.02	0.27	0.00	0.27	0.00	0.01	0.01	0.37
					(1.52							(18.13/18.15)	(18.65/20.23)
Montane	1.37	0.24	0.03	0.00	1.64	0.01	0.12	0.09	0.22	0.21	0.19	20.49	22.35
	0.21	0.07	0.01	0.00	0.16	0.00	0.05	0.00	0.05	0.00	0.02	0.02	0.19
												(20.48/20.52)	(22.22/22.54)

**Figure 2:**
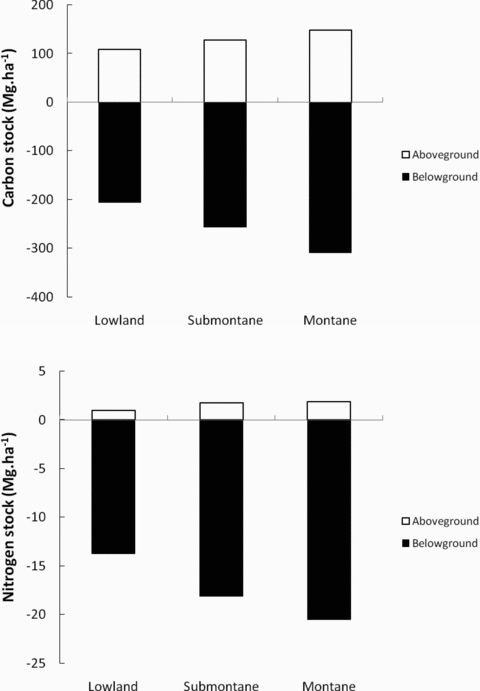
Partitioning of carbon and nitrogen stocks in above- and belowground pools.

Independent of the studied site, C,N*_BGB_* was significantly higher than C,N*_AGB_* (Fig. 2). At 100 m only 35% of the C*_ECOS_* was allocated aboveground, and the remaining 65% was allocated belowground. At higher elevations (1000 m) the ratio C*_ABG_*:C*_ECOS_* decreased to 32%, and consequently, the ratio C*_BGB_*:C*_ECOS_* increased to 68%. For nitrogen, at 100 m only 7% of the N*_ECOS_* was allocated aboveground and the remaining 93% was allocated belowground. The same proportion was maintained at 1000 m (Fig. 2).

### Correlation between carbon and nitrogen stocks and soil temperatures

**Figure 3:**
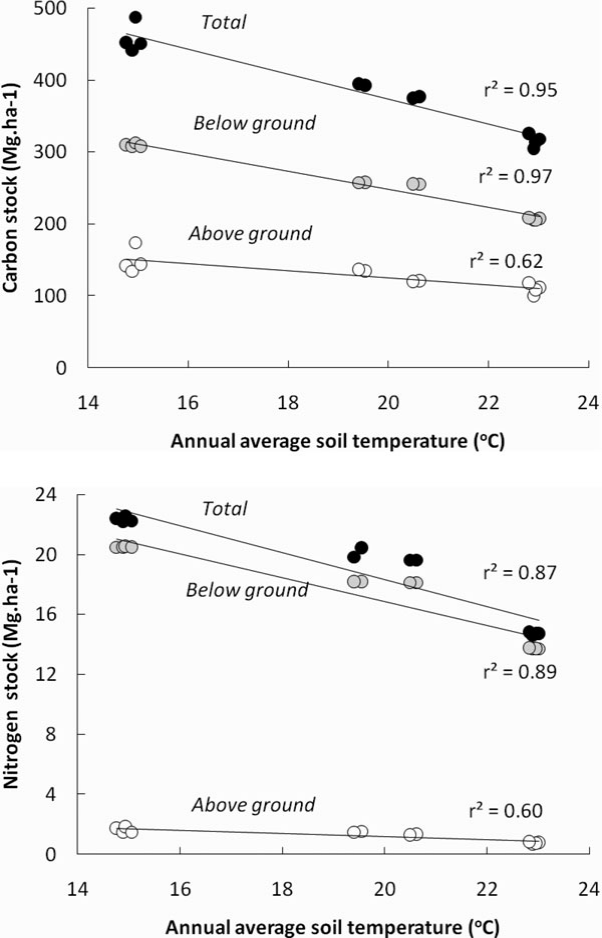
Linear fit to observed data of nitrogen and carbon stocks against soil temperature.

We found significantly inverse correlations between carbon and nitrogen stocks and soil temperatures (Fig. 3). The lowest correlation coefficient for carbon was found between C*_AGLB_* and soil temperature (0.72), increasing to 0.97 and 0.94 between soil temperatures and C*_BGB_* and C*_ECOS_*, respectively (Table 6). For nitrogen, the lowest correlation coefficient was also found between soil temperature and N*_AGLB_* (0.60), increasing again for N*_BGB_* (0.89) and N*_ECOS_* (0.87) (Table 6).

**Table 6:** Parameters estimated for linear regressions between temperature and stocks of carbon and nitrogen. Lower limit (LL) and upper limit (UL) of 95% confidence interval was obtained by bootstrap interaction

					95% Confidence interval		
						
Explained variable	*r*^2^	Parameter	Value	Standard error	LL	UL	*t* statistic (*P*-value)
C_AGB_	0.62	β_0_	187.27	19.22	155.61	221.70	9.79
							(0.0001)
		β_1_	−3.99	0.98	−5.70	−2.29	−4.057
							(0.0022)
C_BGB_		β_0_	497.68	13.099	474.71	522.033	37.99
							(0.0001)
	0.97	β_1_	−12.46	0.67	−13.68	−11.23	−18.61
							(0.0001)
C_ECO_		β_0_	720.84	25.67	674.65	767.17	28.08
							(0.0001)
	0.95	β_1_	−17.38	1.31	−19.80	−14.90	−13.25
							(0.0001)
N_AGB_		β_0_	3.49	0.51	2.67	4.53	6.80
							(<0.0001)
	0.60	β_1_	−0.10	0.026	4.53	−0.058	−3.87
							(0.0031)
N_BGB_		β_0_	32.77	1.77	29.75	35.94	18.55
							(0.0001)
	0.89	β_1_	−0.79	0.090	35.94	−0.64	−8.80
							(0.0001)
N_ECO_		β_0_	36.26	2.13	32.67	40.27	17.02
							(0.0001)
	0.87	β_1_	−0.90	0.11	40.27	−0.71	−8.23
							(<0.0001)

## Discussion

Our main findings were that in all altitudes, more carbon and nitrogen is stored below than aboveground (Fig. 2). Second, the carbon and nitrogen stocks increased with altitude and a significant inverse correlation was found between stocks and average annual soil temperature (Fig. 3).

### Carbon and nitrogen partitioning between above- and belowground along the altitudinal range

It seems that there is a tendency of lowland tropical forests to allocate more carbon aboveground, while in montane forests carbon is preferentially allocated belowground (Kitayma and Aiba 2002; Raich et al. 2006; Girardin et al. 2010). This trend differs from our study sites in two aspects. First, in our study even in the lowland site more carbon is allocated belowground than aboveground (Fig. 2). Second, increases in belowground compared to aboveground carbon stock occur at higher elevations (∼2000—3000 m asl). For instance, in tropical forests of Peru at 1000 m of elevation, most of the carbon was stocked aboveground (60%) than belowground (40%). C*_BGB_* became higher (∼68%) than C*_AGB_* (∼32%) only between 2000 and 3000 m of elevation (Girardin et al. 2010).

Part of the explanation for this difference relies on the fact that above 1000 m C*_AGB_* tends to decrease (Leuschner et al. 2007; Moser et al. 2007), while C*_BGB_* tends to increase (Girardin et al. 2010). In our sites, both C,N*_AGB_* and C,N*_BGB_* increased with elevation, leading to small variations of carbon partitioning between above- and belowground along the elevation range (Fig. 2).

### Temperature and carbon and nitrogen soil storage in the coastal Atlantic Forests

Various studies have reported that the decomposition rate is influenced by the soil and air temperature (e.g., Raich et al. 2006; Wagai et al. 2008). Recently, Dorrepaal et al. (2009) confirmed that increases in temperature could enhance soil carbon losses due to enhanced decomposition and soil respiration (Biasi et al. 2008). Sousa Neto et al. (2011) observed in ours sites a consistent decrease in soil temperature with elevation, and soil and vegetation carbon and nitrogen stocks inversely correlated with mean annual soil temperature in our study (Fig. 3, Table 6).

Our findings have important implications. The Brazilian Atlantic Forest remnants are located mainly on the steepest slopes (Ribeiro et al. 2009), and we found that various aspects of carbon and nitrogen stocks above- and belowground seem to respond to changes in temperature along the elevation range (Table 5). This trend is similar to several studies that have found an increase in soil carbon stocks with an decrease of temperature (e.g., Jobbágy and Jackson 2000; Amundson 2001; Homann et al. 2007). These findings are consistent with findings by Meir and Leuschner (2010) that global change might turn some forest ecosystems into carbon sources, since the increase in forest productivity, expected by the increased in temperature and elevated CO_2_ (Phillips et al. 1998), may not compensate for emissions from the faster mineralization of labile carbon to CO_2_ induced by a warmer climate (e.g., Schlesinger and Andrews 2000).

Our results support a conclusion that climate warming, as is predicted for the southeast region of Brazil (Marengo et al. 2009), may reduce the amount of carbon and nitrogen stored in the coastal Atlantic Forest, turning these ecosystems into carbon and nitrogen sources. In a simple approach, using equations of Table 6, we estimated that an increase of 1°C in mean soil temperature could result in an eventual net transfer of approximately 17 Mg·ha^−1^ (CI 15–20 Mg·ha^−1^)^1^ and 1.0 Mg·ha^−1^ (CI 0–40 Mg·ha^−1^)^1^ of carbon and nitrogen, respectively, from the forest to the atmosphere (Table 5). These figures are significant if we compare with the net primary productivity of Amazon lowland terra-firme forests that varies from approximately 10 to 16 Mg·ha^−1^ per year of carbon (Aragão et al. 2009).

We recognize that these estimates are far too simplistic, since the decomposition rate is not only driven by temperature (Davidson and Janssen 2006), but the patterns reported here can be used in manipulation studies of temperature and precipitation to test these trends and to better understand how ecosystem processes respond to climatic dynamics in tropical forest.

### Comparisons between the carbon and nitrogen stocks in coastal Atlantic Forest and the Amazon Forest

C*_AGLB_* found in the coastal Atlantic Forest varied approximately from 100 to 150 Mg·ha^−1^. This range of values is generally lower than those observed in Amazon lowland *terra-firme* forests located near the municipalities of Manaus, Santarém, and Caxiuanã that have C*_AGLB_* near 200 Mg·ha^−1^ (Rice et al. 2004; Pyle et al. 2008; Malhi et al. 2009); but they were similar to values found for other neotropical forests (Delaney et al. 1997; Clark and Clark 2000; De Walt and Chave 2004).

We believe that the lower C*_AGLB_* found in our sites in relation to Amazon *terra-firme* forests could be related to lower tree density in our sites than other neotropical forests (Delaney et al 1997; Clark and Clark 2000; De Walt Chave 2004; Vieira et al. 2004; Alves et al. 2010). Additionally, the Atlantic Forest has smaller canopy stature than forests in the central, eastern, and southern Amazon (Asner et al., 2002; Nogueira et al., 2008), and what appears to be most important factor, the proportion of big trees (>50-cm DBH) in the Atlantic Forest is lower than that found in Amazon forests with a relatively long dry season (Vieira et al. 2004).

On the other hand, C*_BGB_* were higher in our sites than in tropical lowland and upland forests of the Amazon region (Malhi et al. 2009; Girardin et al. 2010). C*_BGB_* in our site varied from approximately 200 to 300 Mg·ha^−1^, while in lowland forests of the Amazon region C*_BGB_* were lower than 200 Mg·ha^−1^ (Malhi et al. 2009), and in upland forests in the Peruvian Andes were generally lower than 100 Mg·ha^−1^ (Girardin et al. 2010).

There are no data on nitrogen stocks in Amazon forests. For comparisons, we multiplied the *AGLB* found in Manaus and Santarém by Vieira et al. (2004) by the nitrogen concentrations shown in the section Material and Methods for these Amazon forests. N*_AGLB_* was similar in both biomes. On the other hand, N*_BGB_* was approximately double in the Atlantic Forest in relation to the Manaus and Santarém forests.

Several aspects may interfere in below-ground carbon and nitrogen accumulation. Most relevant are climatic variables such as temperature and precipitation (Raich et al. 2002), carbon and nitrogen inputs, vegetation type (Ruiz-Jaen and Potvin 2010; Ushio et al. 2010), litter quality (Hättenschwiler and Jørgensen 2010; Manzoni et al. 2010), and soil characteristics, especially texture (Davidson and Janssen 2006; Zimmermann et al. 2010). Annual rainfall in the coastal Atlantic Forest and in the Amazon region is of the same order of magnitude (>2000 mm). Inputs of carbon and nitrogen via litterfall in the coastal Atlantic Forest are similar to inputs in the Amazon *terra-firme* forests (Sousa Neto et al. 2011). Both sites are dominated by old-growth tropical forests, although species composition are different (Alves et al. 2010). We do not have information yet on litter quality in the coastal Atlantic forests. Therefore, we cannot rule out that differences in litter quality composition may lead to faster decomposition in one area compared to another. Although there is a great deal of variability in soil texture in Amazon forest sites we used for comparisons, soil textures in our sites are not sharply different than soils found in the Amazon sites we used for comparisons (Martins et al. in review). The chemical composition of both soils are also similar, both are poor, acidic tropical soils (Martins et al. in review). We believe that the higher C,N*_BGB_* in soils of the coastal Atlantic Forest in relation to the lowland Amazon forest soils may be explained as a temperature effect (Schlesinger and Andrews 2000; Raich et al. 2002). Even at the lowest site (lowland forest) in the coastal Atlantic Forest, the mean air temperature is lower than in most areas of the Amazon region. It is well established that an increase in the air or soil temperature tends to increase respiration rates (Davidson and Janssen 2006; Dorrepaal et al. 2009; Cusack et al. 2010). Consequently, soils of colder regions tend to lose less carbon to the atmosphere leading to an increase in belowground soil stocks (Raich et al. 2002; Cusack et al. 2010).
